# Technostress, digital fatigue, and AI dependency as antecedents of burnout and SDG-4 achievement in EFL classrooms

**DOI:** 10.1038/s41598-026-45402-7

**Published:** 2026-04-01

**Authors:** Wang Honggang, Abdul Khalique Khoso, Adel R. Althubyani

**Affiliations:** 1 College of International Studies, Yangzhou University, Yangzhou City, China; 2 Department of Curriculum and Educational Technology, College of Education, Taif University, Taif City, Saudi Arabia

**Keywords:** Technostress, Digital fatigue, Sustainable development goals (SDGs), Multigroup analysis (MGA), Technology self-efficacy, Education, Information systems and information technology, Psychology, Psychology

## Abstract

This study examines the impact of digital stressors (technostress, digital fatigue, AI dependency) on digital burnout and Sustainable Development Goal (SDG) achievement among Chinese and international EFL learners, investigating the mediating roles of foreign language anxiety and the moderating effect of technology self-efficacy. Using a cross-sectional survey design with *n* = 545 university students, we employed structural equation modeling (SEM) and multigroup analysis (MGA) to test the hypothesized relationships. The findings confirm a significant sequential pathway where digital stressors increase foreign language anxiety, which subsequently exacerbates digital burnout, ultimately impairing SDG achievement. Crucially, technology self-efficacy served as a vital buffer, significantly weakening the negative impact of burnout on learning outcomes. Multigroup analysis revealed these effects were significantly stronger for international students, who also demonstrated higher vulnerability to digital stressors but benefited more from self-efficacy. The study’s novelty lies in its integrated model that uncovers the psychological mechanisms translating digital challenges into educational outcomes, while highlighting crucial cross-cultural variations. These findings offer imperative implications for developing tailored pedagogical interventions that leverage self-efficacy training and differentiated support systems to foster more equitable and sustainable digital learning ecosystems for diverse student populations.

**Supplementary Information:**

The online version contains supplementary material available at 10.1038/s41598-026-45402-7.

## Introduction

The pervasive integration of digital technologies, such as artificial intelligence (AI), online platforms, and digital assessments, has fundamentally reshaped foreign language education^1–3^. While these tools enable personalized and accessible learning, a growing body of literature documents their concomitant psychological challenges, including technostress^4,5^, digital fatigue^6^, and concerns regarding over-reliance on AI systems^7^. These digital stressors are of particular concern in English as a Foreign Language (EFL) contexts, where they may interact with established affective barriers like Foreign Language Learning Anxiety (FLLA)^8,9^. Building on this foundation, the present study integrates these research streams to propose and test a sequential model of psychological strain in digital learning environments.

Drawing on the logic of resource conservation theories (Hobfoll 1989), we posit that digital stressors conceptualized here as the combined experience of technostress, digital fatigue, and perceived AI dependency deplete learners’ emotional and cognitive resources. This depletion is theorized to intensify pre-existing language-specific anxieties, creating a compounded affective burden. In turn, this heightened FLLA is hypothesized to act as a critical mediator, fueling a state of digital burnout, a syndrome of exhaustion, cynicism, and reduced efficacy linked to technology use in academic settings^10,11^. This progression from digital stressors to anxiety to burnout represents a significant threat to the core objectives of Sustainable Development Goal 4 (SDG-4), which advocates for inclusive, equitable, and quality education (Al-Smadi et al. 2024)^54^^12^. When psychological strain impairs engagement and well-being, the sustainability of educational outcomes is compromised^13^.

Thus, this research extends current understanding by investigating three specific, interrelated gaps. First, while digital stressors and FLLA have often been studied in isolation, their synergistic effect remains underexplored^14^. Second, the mediating role of FLLA in the pathway linking digital stressors to digital burnout requires empirical validation. Third, the consequences of this psychological progression for the perceived attainment of sustainable education goals (SDG-4) are largely unexamined. Furthermore, the study investigates technology self-efficacy as a potential moderating buffer against this negative chain of influence. This study does not examine isolated phenomena but tests an integrated model that connects digital stressors, FLLA, and digital burnout within the overarching framework of sustainable education. By doing so, it aims to provide a nuanced understanding of how the psychological costs of digital learning environments can undermine educational quality and equity, while also identifying potential protective factors for educators and policymakers to leverage.

The theoretical contributions of this study are threefold. First, it advances foreign language anxiety research by shifting the focus from conventional classroom triggers to digital-specific stressors, extending Horwitz et al.’s^9^ foundational work into the digital age. Second, it enriches the technostress and digital burnout literature by applying these concepts to EFL contexts, where language acquisition’s cognitive and emotional demands may intensify digital stress. Third, it contributes to the emerging discourse on AI ethics in education by empirically testing how AI dependency influence learner anxiety and burnout, a critical yet overlooked dimension in AI adoption studies. By situating these relationships within the broader framework of SDG-4, this study also underscores the societal relevance of mitigating digital stress to promote equitable and sustainable education. From a practical standpoint, this research offers actionable insights for educators, policymakers, and EdTech developers.

### Theoretical background

This study is guided by an integrative theoretical framework combining insights from Technostress Theory, the Job Demands-Resources (JD-R) model, and Control-Value Theory (CVT). This synthesis provides a coherent explanatory pathway for how specific digital stressors lead to psychological strain, which in turn undermines sustainable educational outcomes aligned with SDG-4. Technostress Theory^15,16^ forms the foundation by directly defining and operationalizing the primary antecedents in our model: the digital stressors of techno-overload, techno-complexity, and techno-insecurity (extended here to include fears of AI replacement). These are conceptualized as specific, negative demands placed on learners in technology-infused environments.

The psychological process through which these technical demands translate into emotional outcomes is informed by Control-Value Theory (CVT)^17^. CVT provides the critical conceptual link, proposing that achievement emotions like anxiety arise from an individual’s appraisals of control over activities/outcomes and the subjective value of those outcomes. In our context, we posit that the digital stressors identified by Technostress Theory (e.g., confusing interfaces, system failures, pressure to master AI tools) threaten learners’ perceived control over the learning process. Simultaneously, the high-stakes nature of language proficiency and algorithmic evaluations can heighten the subjective value of outcomes.

The Job Demands-Resources (JD-R) model^18^ then explains the subsequent progression from acute stress and anxiety to a chronic, debilitating state. It frames digital stressors and the ensuing FLLA as cumulative psychological demands that deplete learners’ emotional and cognitive energy. Prolonged exposure to these demands is theorized to lead to digital burnout a state of exhaustion, cynicism, and reduced efficacy. Furthermore, the JD-R model provides the theoretical basis for including technology self-efficacy as a key personal resource that can buffer the impact of demands, helping to explain variance in individual resilience. Finally, the framework is extended into the domain of educational sustainability by linking this psychological progression to the attainment of Sustainable Development Goal 4 (SDG-4). Digital burnout systematically impairs the pillars of SDG-4: it diminishes quality through cognitive exhaustion, threatens equity by disproportionately affecting less confident or resource-poor learners, and undermines inclusion by creating psychological barriers to participation.

## Literature review

### Perceived AI dependency, digital fatigue and technostress

The proliferation of digital tools in educational settings, while enhancing accessibility and personalization, has introduced a suite of psychological stressors that uniquely impact language learners^19^, Alhur et al., 2025). Central among these is technostress, a multidimensional construct arising from an individual’s inability to adapt to or cope with the demands of technology use^15^. In language learning contexts, this manifests through techno-overload from constant digital demands and techno-complexity from navigating multifunctional platforms (Zivi et al., 2025), which can overwhelm cognitive resources and induce significant mental strain^4^. This strain is posited to directly undermine the emotional security required for language acquisition, exacerbating the situation-specific apprehensions that characterize Foreign Language Learning Anxiety (FLLA)^20^. Building on this established link between technological strain and general academic anxiety.

The digital learning environment also fosters two interrelated yet distinct stressors: digital fatigue and perceived AI dependency^21^. Digital fatigue, characterized by cognitive and emotional exhaustion from prolonged screen-based engagement (Almutairi, 2025), depletes the very mental energy required for the active processing and social risk-taking inherent in language practice^6, Prasetyo et al., 2025)^. This state of depletion is theorized to lower emotional resilience, making learners more vulnerable to anxiety-provoking aspects of language performance^22^. Concurrently, perceived AI dependency the sense of reliance on AI tools for learning tasks can paradoxically evoke anxiety through fears of deskilling, erosion of learner autonomy, and pressure to conform to opaque algorithmic assessments^7, Lin & Yu, et al., 2025)^. This perception threatens core psychological needs for competence and control, creating fertile ground for anxiety^12^. These three digital stressors are thus conceptualized as interconnected antecedents that collectively amplify the affective barriers to successful language acquisition^23, (Bourlakis et al., 2023)^. Therefore, it is further hypothesized that:

#### H_1_

Technostress has a significant positive effect on Foreign Language Learning Anxiety.

#### H_2_

Digital Fatigue has a significant positive effect on Foreign Language Learning Anxiety.

#### H_3_

Perceived AI Dependency has a significant positive effect on Foreign Language Learning Anxiety.

### Digital burnout and sustainable development goal 4 (SDG-4)

The progression from digital stressors and anxiety to more chronic psychological impairment is conceptualized through the lens of digital burnout^24^. Extending the well-established Job Demands-Resources (JD-R) model^18^ to the academic domain, digital burnout is characterized by emotional exhaustion, depersonalization (or cynicism towards learning), and a reduced sense of personal accomplishment related to one’s studies^10,11^. Within this framework, sustained psychological demands such as the persistent apprehension and cognitive load of Foreign Language Learning Anxiety (FLLA) are theorized to deplete students’ emotional and motivational resources over time^25^. When learners face chronic anxiety without adequate recovery or coping resources, this depletion can culminate in the syndrome of burnout, marking a shift from an acute stress response to a state of chronic academic disengagement and weariness^26^. Thus, FLLA is posited not merely as a concurrent stressor but as a critical mediator that channels the effects of various digital demands into this debilitating outcome^27^.

This escalation of psychological strain has profound implications for the sustainability of education, directly threatening the aims of Sustainable Development Goal 4 (SDG-4), which promotes inclusive, equitable, and quality lifelong learning^1,2,28^. To bridge the macro-level goal with individual experience, this study operationalizes SDG-4 achievement as learners’ perceived attainment of these pillars within their immediate educational environment encompassing their appraisal of instructional quality, the fairness of opportunities (equity), and the sense of belonging and support (inclusion)^29^. Digital burnout systematically undermines each of these dimensions: exhaustion impairs cognitive engagement and perceived learning quality, cynicism fosters disengagement and threatens equity by creating psychological barriers to participation; and a diminished sense of efficacy compromises the inclusive ideal of education for all^30^. Consequently, digital burnout is framed not as an isolated personal outcome, but as a key mechanism that erodes the foundational conditions required for sustainable educational development, thereby creating a direct pathway from micro-level distress to macro-level goal impairment^31^. Based on above literature following hypotheses are proposed:

#### H_4_

Foreign Language Learning Anxiety has a significant positive effect on Digital Burnout.

#### H_5_

Digital Burnout has a significant negative effect on the perceived achievement of Sustainable Development Goal 4 (SDG-4).

### Moderating role technology self-efficacy

Amidst the documented negative psychological progression from digital stressors to burnout, personal resources can serve as critical buffers that alter the impact of these demands^32^. Technology self-efficacy an individual’s belief in their capability to use technology effectively to accomplish specific tasks is one such pivotal resource rooted in Social Cognitive Theory (Bandura 1986)^56^. In the context of the Job Demands-Resources (JD-R) model, self-efficacy functions as a key personal resource that can mitigate the depleting effects of job demands (or, in this case, academic demands) on wellbeing and performance^18^ (Xanthopoulou et al. 2007)^55^. Learners with high technology self-efficacy are theorized to appraise technological challenges as manageable rather than threatening (Pan, 2020), employ more adaptive coping strategies, and persist longer in the face of digital difficulties (Compeau and Higgins 1995)^57^. Consequently, this resource may not only reduce the initial development of digital burnout but, crucially, may also weaken the strength of the relationship between established burnout and its deleterious outcomes^33^. Specifically, for students experiencing digital burnout, a strong sense of technology self-efficacy could provide a psychological counterweight, helping to preserve their perception of educational quality and engagement (Honarzad & Rassaei, 2019), thereby attenuating the negative impact of burnout on their perceived achievement of SDG-4's goals of quality, equity, and inclusion^34^. Thus, it is hypothesized that:

#### H_6_

Technology self-efficacy significantly moderates the relationship between Digital Burnout and SDG-4 achievement, such that the negative effect of burnout is weaker for learners with higher levels of technology self-efficacy.

### Mediating role of foreign language learning anxiety (FLLA)

**Fig. 1:**
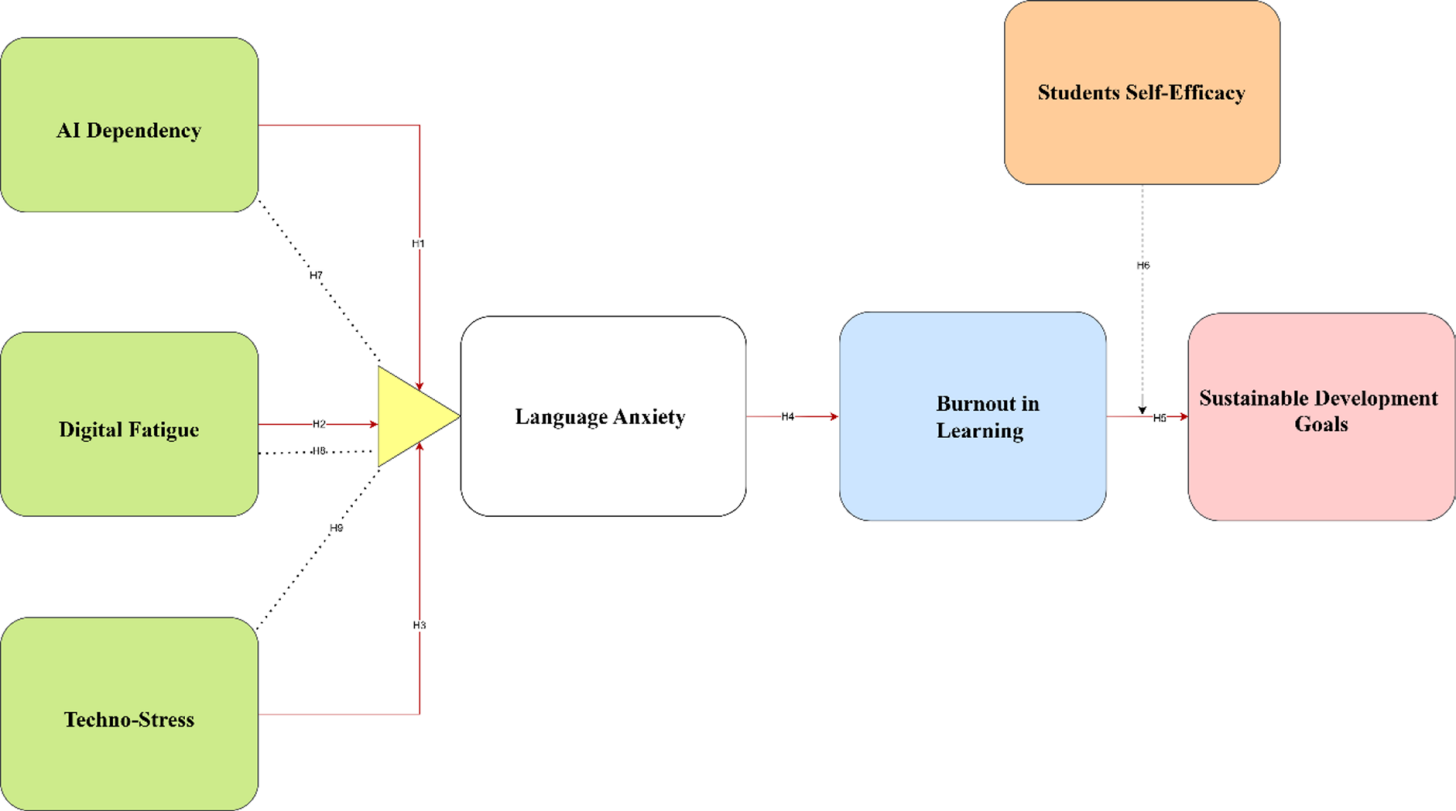
Conceptual Framework.

The psychological pathway from digital stressors to the chronic syndrome of digital burnout is unlikely to be direct; rather, it is theorized to operate through the amplification of situation-specific affective states^35^. Foreign Language Learning Anxiety (FLLA) is a well-established construct that captures the unique apprehension, worry, and tension associated with learning and using a second language^9,36^. This anxiety acts as a critical mechanism that channels broader digital stress into the academic domain. Specifically, the demands of technostress^4^, the exhaustion from digital fatigue^6^, and the apprehension tied to perceived AI dependency^7^ are each posited to heighten learners’ anxiety in the language classroom by threatening their sense of control, competence, and emotional security. This elevated, persistent anxiety, in turn, functions as a continuous and draining psychological demand. According to the conservation of resources theory (Hobfoll 1989)^53^, sustaining this anxious state depletes emotional and cognitive energy over time, creating the exhaustion and diminished efficacy that define digital burnout^10^. Therefore, FLLA is not merely a concurrent outcome of digital stressors but is hypothesized to be the pivotal mediating process that explains how these initial technological pressures culminate in a state of chronic academic burnout (Fig. 1). Consequently, it is posited that:

#### H_7_

Foreign Language Learning Anxiety significantly mediates the relationship between Technostress and Digital Burnout.

#### H_8_

Foreign Language Learning Anxiety significantly mediates the relationship between Digital Fatigue and Digital Burnout.

#### H_9_

Foreign Language Learning Anxiety significantly mediates the relationship between Perceived AI Dependency and Digital Burnout.

## Methodology

### Research design

This study employs a quantitative, cross-sectional research design to examine the relationships between digital stressors (technostress, digital fatigue, and perceived AI dependency), foreign language learning anxiety (FLLA), digital burnout, and the perceived achievement of SDG-4 in an EFL context. The study’s primary objective is to test a complex structural model that incorporates predictive relationships, mediation, and moderation effects. Given this predictive and exploratory focus, the research design utilizes partial least squares structural equation modeling (PLS-SEM) as the primary analytical method. PLS-SEM was selected over covariance-based SEM (CB-SEM) for three key reasons: first, its suitability for modeling complex, hierarchical path models even with multiple mediators; second, its ability to maximize the explained variance of the ultimate dependent variable (SDG-4 attainment); and third, its robustness with non-normally distributed data, which is common in psychological and perceptual measures. This approach is ideal for capturing the complex interplay of variables within the proposed theoretical framework and for analyzing both direct and indirect pathways in a single, integrated model.

### Participants and sampling

This study targeted university-level EFL learners engaged in technology-mediated language learning across four major public universities in China. The final sample comprised N = 545 participants, which included n = 412 Chinese domestic EFL students and n = 133 international students enrolled in English-taught programs. The international student group was defined as non-native English speakers pursuing degree programs in China, representing diverse linguistic and cultural backgrounds (e.g., from Southeast Asia, Africa, and the Middle East) to facilitate cross-cultural comparison. Participants were recruited via a stratified random sampling approach, with stratification based on three criteria to ensure representativeness: (1) English language proficiency level (beginner, intermediate, advanced), as determined by their institution’s placement tests or self-reported CEFR-equivalent levels; (2) academic discipline (Applied Linguistics, English Language Teaching, and Literature); and (3) self-reported frequency of digital tool usage in learning (low, moderate, high). All participants, regardless of nationality, completed the same English-language version of the questionnaire to maintain consistency in measurement, as the study context was English-medium instruction. A G*Power analysis confirmed sample size adequacy, indicating that N = 545 provided > 95% statistical power to detect medium effect sizes (f^2^ = 0.15) in structural equation modeling at α = 0.05. The inclusion of international students allowed for the examination of potential cultural or linguistic moderators in the digital stress pathway, addressing a gap in cross-cultural technostress research.

Table 1 substantiates the successful implementation of the stratified random sampling strategy. The sample demonstrates balanced representation across the predefined strata, including English proficiency levels, academic disciplines related to language study, and frequency of digital tool usage. This balanced stratification enhances the ecological validity of the findings and supports the robustness of subsequent subgroup analyses. The proportional inclusion of both Chinese (75.6%) and international students (24.4%) further ensures that the model is tested across diverse learner backgrounds as intended.

**Table 1:** Participant Demographics and Stratification (N = 545).

Stratification characteristic	Category	Frequency (n)	Percentage (%)
Student Status	Chinese EFL Students	412	75.60
	International Students	133	24.40
English Proficiency Level	Beginner (A2)	98	18.00
	Intermediate (B1-B2)	321	58.90
	Advanced (C1 +)	126	23.10
Academic Discipline	Applied Linguistics	187	34.30
	English Language Teaching (ELT)	204	37.40
	English Literature	154	28.30
Frequency of Digital Tool Use	Low	102	18.70
	Moderate	279	51.20
	High	164	30.10
Gender	Female	328	60.20
	Male	217	39.80
Academic Year	Undergraduate (Years 1–2)	245	45.00
	Undergraduate (Years 3–4)	193	35.40
	Postgraduate	107	19.60

### Data collection procedures

The data collection procedures were meticulously designed to ensure methodological rigor and cross-cultural validity. Following ethical approval from the institutional review board, all measurement instruments underwent a comprehensive adaptation process to ensure their appropriateness for the context of this study. A pilot study (n = 45) was subsequently conducted, which confirmed strong reliability for all scales (Cronbach’s α > 0.82) and established unidimensionality through confirmatory factor analysis with all factor loadings exceeding 0.70. The main data collection was administered through both online and paper-based surveys across the four participating universities, with trained research assistants present to ensure procedural consistency and address participant queries. Prior to analysis, all collected data underwent rigorous screening for missing values, outliers, and response patterns, with the final dataset demonstrating excellent psychometric properties that confirmed the adapted instruments effectively captured the target constructs within the specific cultural and educational context of this investigation.

**Fig. 2:**
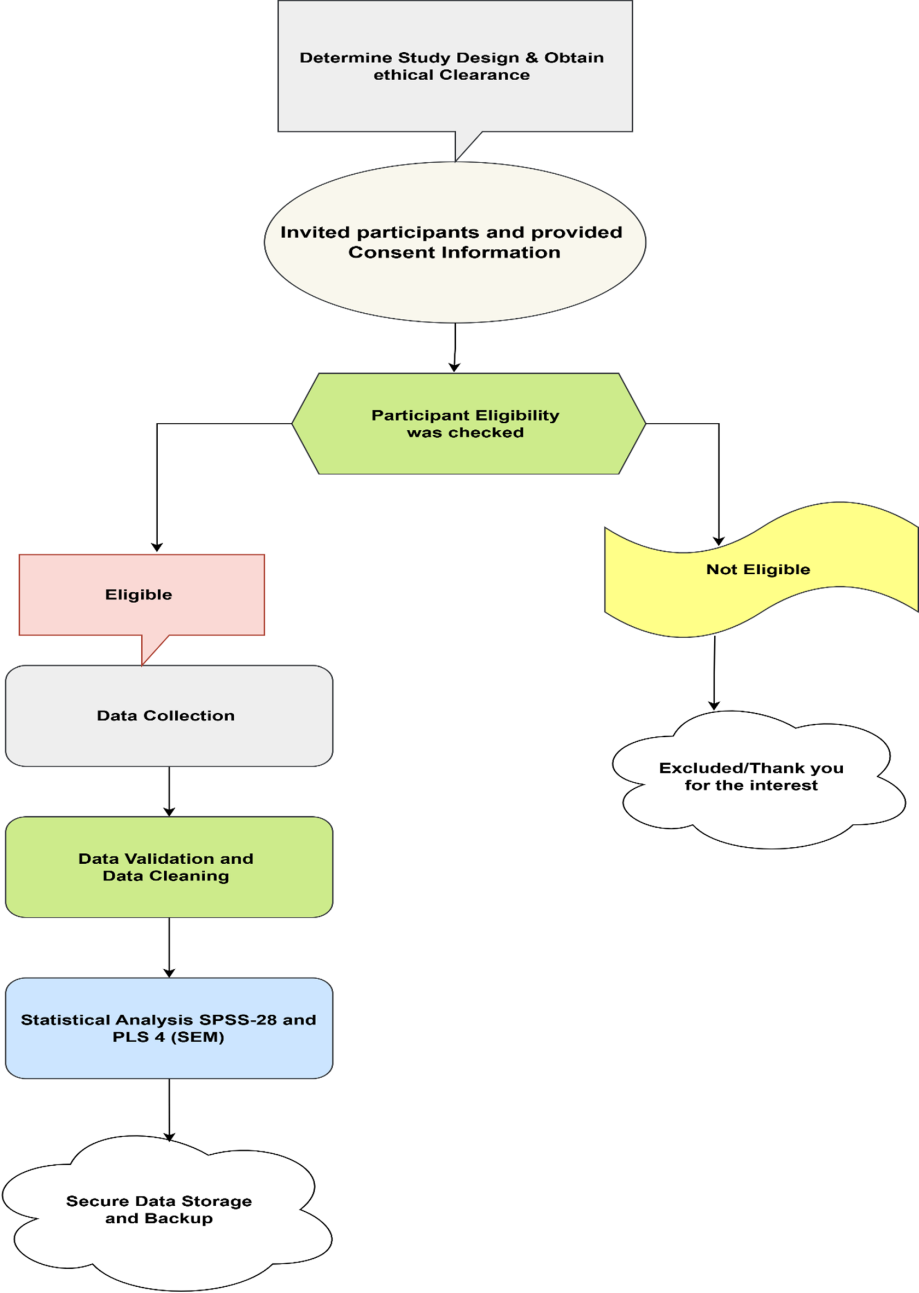
Data Collection Flowchart.

Figure 2 below shows the data collection process, which visually represents the data collection process in sequential steps. Flowcharts are a powerful way to illustrate the path from initial participant recruitment through data screening and analysis, as they provide a top-down view of each stage in the workflow.

### Data collection instruments

#### Technostress

Technostress was measured using a validated scale adapted from Wang et al.^37^, comprising two distinct subscales: (1) Abilities-Demands Misfit (ADT), a 7-item subscale assessing the perceived gap between students’ technological competence and the demands of digital learning tools, and (2) Needs-Supplies Misfit (NST), a 6-item subscale evaluating discrepancies between learners’ expectations and the actual institutional support provided for technology integration. This bifurcated approach captures both the internal (skill-based) and external (resource-based) dimensions of technostress, aligning with the study’s focus on how systemic and personal factors jointly contribute to digital stress in EFL contexts. The scale’s reliability (α > 0.85 in prior studies) and construct validity make it suitable for examining technostress in higher education settings.

#### Digital fatigue

Digital fatigue was assessed using a 6-item scale adapted from Sarangal and Nargotra^38^, designed to measure students’ cognitive and emotional exhaustion from prolonged engagement with digital learning platforms. The scale showed reliability (c < 0.80 in prior analysis) and resonates with the current findings in the literature that revolves around the technology-related exhaustion levels in the education field. The adaptation that was done in this study consisted of slight linguistic adjustments to make the scale of interest culturally suitable to the EFL learner population and preserve the psychometric properties of the original scale.

#### Perceived AI dependency

This study employed the multidimensional scale developed by Morales-García et al.^39^ to assess students’ Perceived AI Dependency in education, this scale has 5 items. The instrument has demonstrated strong psychometric properties in previous studies (α = 0.87–0.93 across subscales) and was particularly suitable for this investigation given its focus on educational technology contexts.

#### Foreign language learning anxiety (FLLA)

Foreign language learning anxiety was measured using a validated scale adapted from Briesmaster and Briesmaster-Paredes^40^, which assesses three distinct dimensions of language-related anxiety: Communicative Apprehension (11 items), measuring discomfort in speaking and listening situations, Test Anxiety (15 items), evaluating stress related to language assessments and evaluations; and Fear of Negative Evaluation (7 items), capturing concerns about being judged negatively by peers or instructors. This multidimensional 33 item tool effectively measures psychological barriers involved in language acquisition, especially in the digital learning setting. It has been found to be highly reliable (alpha 0.85 or above in the past studies) across scales and was contextualized to modern day language learning situations.

#### Digital burnout in learning

The measurement of digital burnout was conducted using a validated scale adapted from Erten and Özdemir^41^, which captures three critical dimensions of technology-related exhaustion in educational settings: Digital Aging (12 items), assessing the perceived obsolescence of digital skills and technological fatigue,Digital Deprivation (6 items), measuring the psychological impact of technology overuse and the need for digital detoxification; and Emotional Exhaustion (6 items), evaluating feelings of depletion and reduced motivation due to prolonged digital engagement. It is a 24-item dimension that examines, at length, the negative outcomes of extended technology use in the learning environment. The scale has proven reliable (0.85 across subscales in previous works) and was chosen due to its applicability to the present-day digital learning environment.

#### Sustainable development goals (SDG) achievement

The perceived attainment of SDG-4 (Quality Education) at the individual learner level was measured using an adapted version of the Sustainable Development Awareness scale by Atmaca et al.^42^. This instrument was selected because its sub-dimensions correspond directly to the core pillars of SDG-4: 1) Social Sustainability (9 items) was used to measure perceptions of equity and inclusiveness, 2) Economic Sustainability (12 items) was adapted to assess perceived educational quality and value; and 3) Environmental Sustainability (14 items) was included to capture the broader context of sustainability-oriented education. For this study, a confirmatory factor analysis validated a three-factor structure consistent with these dimensions. The scale demonstrated high internal consistency, with Cronbach’s alpha values for the subscales all exceeding 0.85 in the present sample.

#### Technology self-efficacy

The measurement of technology self-efficacy was conducted using the validated multidimensional scale developed by Tsai et al.^43^, which assesses five distinct dimensions of learners’ confidence in utilizing digital tools for educational purposes: Practice (4 items), measuring the ability to use technology for learning consistently, Application (3 items), evaluating skills in implementing technology for specific tasks; Collaboration (3 items), assessing competence in using digital tools for group work; Comprehension (3 items), gauging understanding of technological concepts; and Analysis (3 items), examining the ability to evaluate digital information critically.

### Data analysis

Data were analyzed using a two-phase approach in SPSS (v.28) and SmartPLS (v.4.0). Preliminary analyses in SPSS included descriptive statistics, reliability assessment (Cronbach’s alpha), and bivariate correlations. Structural equation modeling was conducted in SmartPLS (PLS-SEM) to test the hypothesized model. The analysis followed a standard two-step procedure: (1) assessment of the measurement model to confirm reliability, convergent validity (outer loadings, AVE), and discriminant validity (HTMT criterion); and (2) evaluation of the structural model for path coefficients, significance (via 5,000 bootstrap samples), and explanatory power (R^2^ values). The study’s predictive focus was reinforced by assessing predictive validity (Q^2^ via blindfolding) and out-of-sample predictive power (PLS-predict). In line with best practice for PLS-SEM, model fit was evaluated using the Standardized Root Mean Square Residual (SRMR), with the value of 0.063 indicating a good overall fit to the data. Furthermore, robustness checks included Harman’s single factor test for common method bias and a multigroup analysis (MGA) to test for significant differences in path coefficients between Chinese and international student subgroups. This comprehensive analytical strategy ensured rigorous testing of both measurement quality and the proposed theoretical pathways.

### Ethical considerations

This study adhered to strict ethical protocols. Prior to data collection, this study was reviewed and approved by the Institutional Review Board (IRB) of Yangzhou University (Academic Committee of the College of International Studies). All participants were provided with a detailed information sheet outlining the study’s purpose, procedures, and their rights, and informed consent was obtained electronically before proceeding. Participation was entirely voluntary, with the right to withdraw at any point without consequence. Data were collected anonymously, with no personally identifiable information recorded, and were stored securely on password-protected servers to ensure confidentiality. The research design posed no more than minimal risk to participants.

## Results

### Descriptive statistics

The descriptive statistics provide an initial overview of the key variables in the study, offering insights into participants’ experiences with digital stressors, language learning anxiety, and academic performance. The means, standard deviations, and reliability coefficients for all constructs are presented in Table 2.

**Table 2:** Descriptive Statistics.

Variable	Mean	SD	Skewness	Kurtosis	Min.	Max.
Technostress	3.38	0.84	−0.24	0.32	1.0	5.00
Digital Fatigue	3.62	0.79	−0.31	0.28	1.0	5.00
Perceived AI Dependency	3.65	0.77	−0.29	0.41	1.0	5.00
Foreign Language Anxiety	3.53	0.82	−0.26	0.35	1.0	5.00
Digital Burnout	3.41	0.81	−0.18	0.22	1.0	5.00
Technology Self-Efficacy	3.91	0.74	−0.41	0.55	1.0	5.00
SDG Achievement	3.72	0.71	−0.33	0.48	1.0	5.00

Table 2 presents the descriptive statistics for all study variables, which were measured on a 1-to-5 Likert scale. The mean scores ranged from 3.38 (Technostress) to 3.91 (Technology Self-Efficacy), with standard deviations varying between 0.71 (SDG Achievement) and 0.84 (Technostress). The analysis of distribution characteristics showed that all variables demonstrated negative skewness, with values from −0.41 (Technology Self-Efficacy) to −0.18 (Digital Burnout), and positive kurtosis, with values ranging from 0.22 (Digital Burnout) to 0.55 (Technology Self-Efficacy). The actual data range for all variables spanned the entire possible scale from the minimum of 1.00 to the maximum of 5.00.

The moderately high mean scores for SDG-Achievement (M = 3.72) and Technology Self-Efficacy (M = 3.91) could reflect a genuinely positive perception of the learning environment and students’ confidence in their digital skills. However, the ceiling tendency, where most scores cluster above the scale midpoint, may also indicate a potential self-report positivity bias common in perceptual surveys, where respondents might overstate positive attributes due to social desirability.

**Fig. 3:**
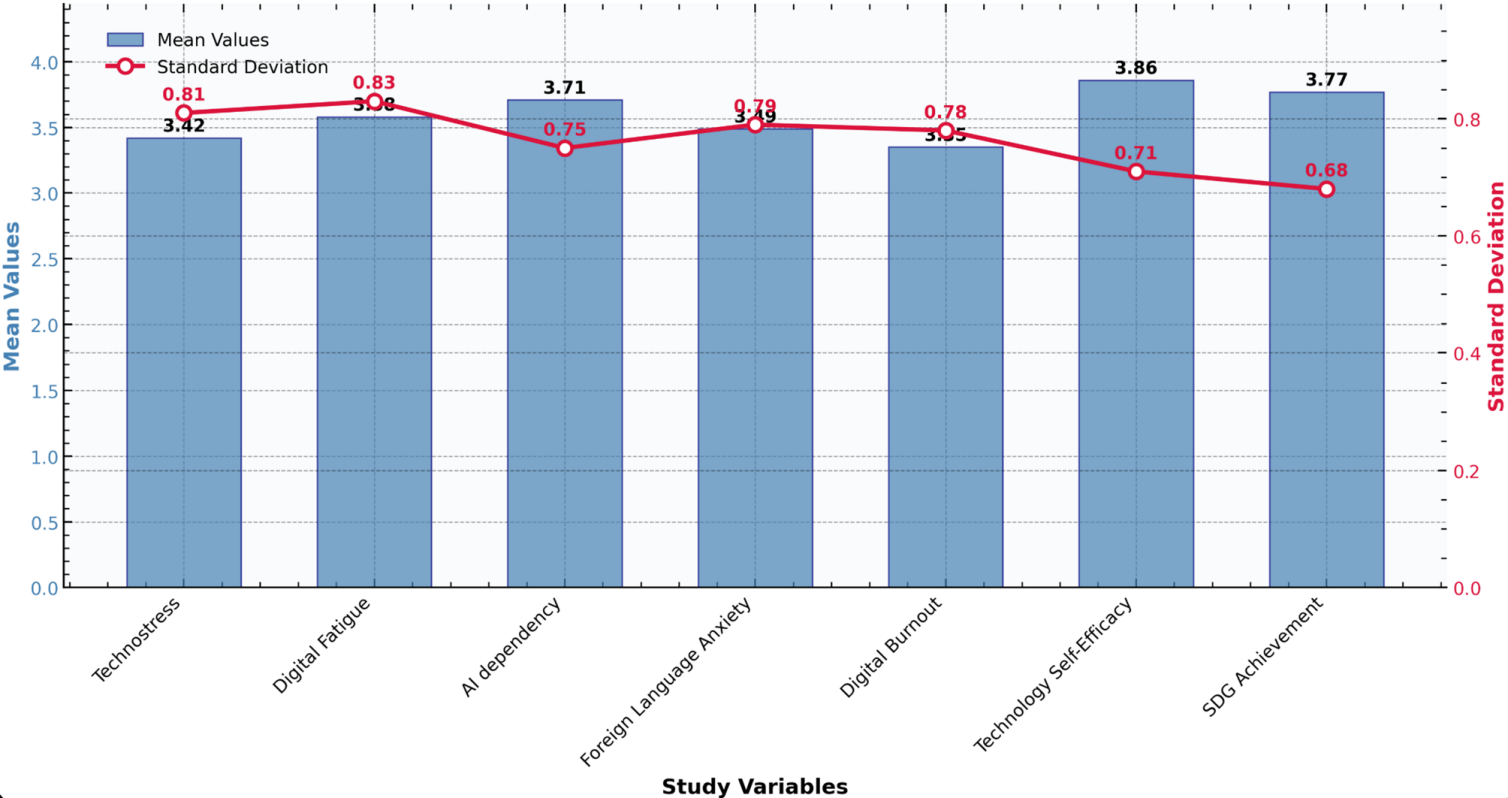
Descriptive Statistics of Study Variables.

Figure 3 shows the numerical values for each variable are as follows: Digital Fatigue (Mean ≈ 3.62), AI Dependency (Mean ≈ 3.65), Foreign Language Anxiety (Mean ≈ 3.53), Digital Burnout (Mean ≈ 3.41), and Technology Self-Efficacy (Mean ≈ 3.91). The corresponding standard deviations for these variables are approximately 0.79, 0.77, 0.82, 0.81, and 0.74, respectively.

Table 3 reveals a coherent pattern where the negative psychological states Technostress, Digital Fatigue, Foreign Language Anxiety, and Digital Burnout are all strongly and positively interlinked, suggesting they form a cluster of related distress in the digital learning environment. Conversely, Technology Self-Efficacy acts as a protective factor, demonstrating significant negative correlations with these adverse outcomes while simultaneously showing a strong positive correlation with the desired outcome of SDG Achievement.

**Table 3:** Correlation Matrix of Study Variables.

Variable	1	2	3	4	5	6	7
1. Technostress	1						
2. Digital Fatigue	0.68**	1					
3. AI Dependency	0.42**	0.38**	1				
4. Foreign Language Anxiety	0.51**	0.55**	0.29**	1			
5. Digital Burnout	0.71**	0.75**	0.35**	0.58**	1		
6. Technology Self-Efficacy	−0.41**	−0.38**	0.15*	−0.33**	−0.45**	1	
7. SDG Achievement	−0.35**	−0.31**	0.22**	−0.28**	−0.39**	0.52**	1

### Common method variance (CMV) assessment

Given that the study relied on self-reported data collected through a single survey, potential common method variance (CMV) was assessed using both procedural and statistical controls. Procedurally, we ensured respondent anonymity, varied scale endpoints, and randomized item order to minimize bias.

Table 4 shows assessment of common method bias indicates that it is not a substantial threat to the validity of the study’s findings. Harman’s single-factor test revealed that a single factor accounted for only 31.4% of the variance, which is well below the critical threshold of 50%. Furthermore, while the correlation matrix contains a high value of 0.75, this relationship is theoretically sound and does not suggest an artificial inflation of relationships due to the measurement method. Therefore, the results can be interpreted with confidence that they reflect genuine variable relationships rather than methodological artifacts.

**Table 4:** Common Method Bias Assessment.

Statistical Test / Indicator	Result	Interpretation
Harman’s Single-Factor Test	31.40%	The total variance explained by a single factor is below the 50% threshold, indicating that common method bias is not a critical concern in the data
Highest Correlation in Matrix	0.75 (Digital Fatigue & Digital Burnout)	While high, this correlation is not excessively high (e.g., > 0.90) and is theoretically justified, suggesting the relationship is substantive and not merely an artifact of common method variance

### Measurement model results

Establishing the validity and reliability of the measurement model is a critical prerequisite for testing the structural model, as it confirms that the latent constructs are accurately measured by their assigned indicators and that the model fits the observed data well. This step ensures that subsequent findings and conclusions about the relationships between variables are built upon a psychometrically sound foundation.

**Fig. 4:**
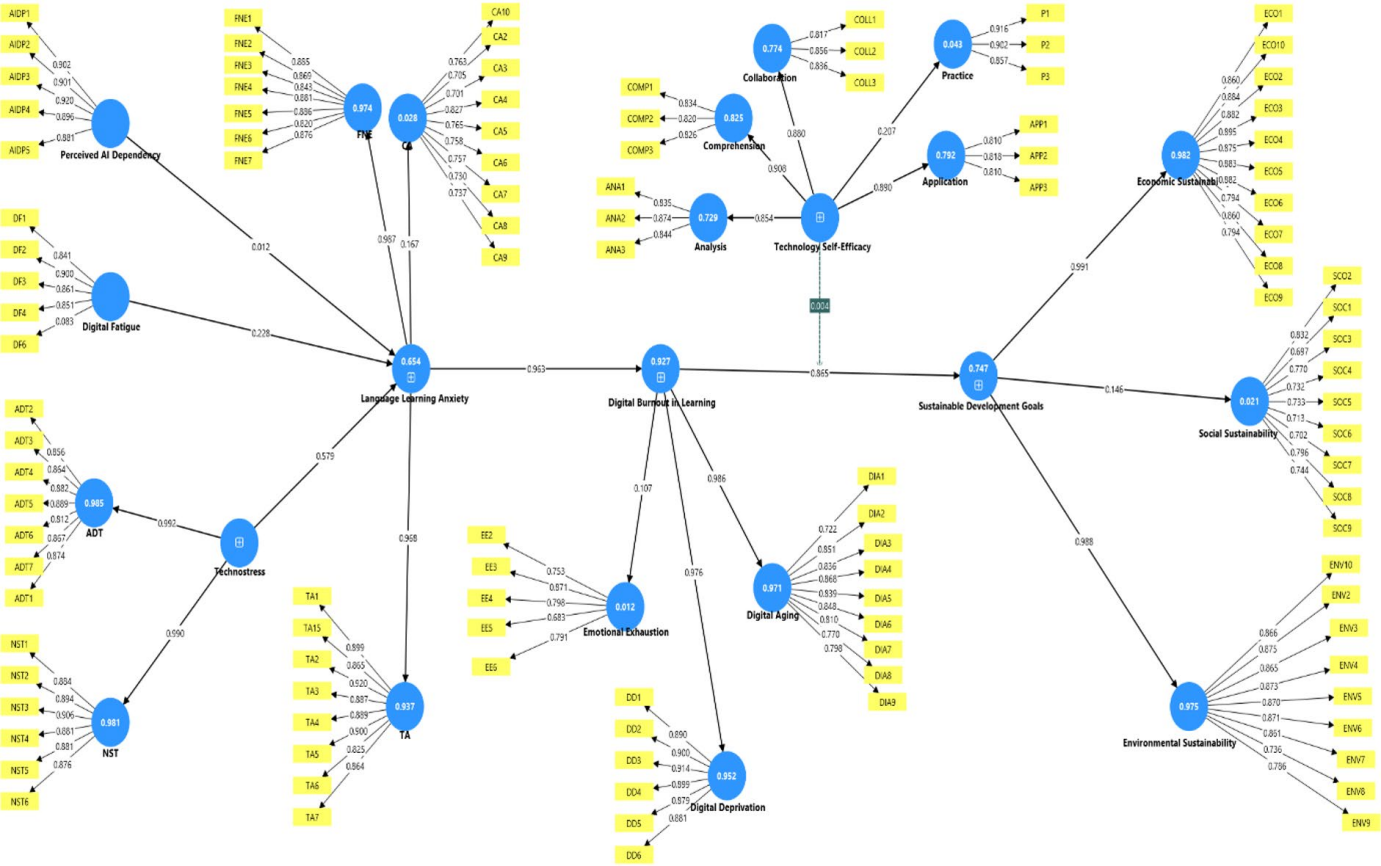
Final Measurement Model.

Table 5, demonstrate that all constructs met the established criteria for reliability and convergent validity. All standardized factor loadings exceeded the recommended threshold of 0.70, ranging from 0.78 (NST dimension of Technostress) to 0.90 (Digital Deprivation dimension of Digital Burnout). Composite reliability values for all constructs ranged from 0.88 (Perceived AI Dependency) to 0.94 (Technology Self-Efficacy), exceeding the 0.70 threshold and indicating excellent internal consistency. The Average Variance Extracted values ranged from 0.62 (Technostress) to 0.77 (Digital Fatigue and Sustainable Development Goals), all surpassing the recommended 0.50 criterion. Similarly, Cronbach’s Alpha coefficients ranged from 0.86 (Perceived AI Dependency) to 0.93 (Technology Self-Efficacy), further confirming the reliability of the measurement instruments (Fig. 4).

**Table 5:** Measurement Model Results.

Variable & dimensions	Composite reliability (CR)	Average variance extracted (AVE)	Cronbach’s Alpha (α)
1. Technostress	0.89	0.62	0.87
ADT			
NST			
2. Digital Fatigue	0.91	0.77	0.9
3. Perceived AI Dependency	0.88	0.71	0.86
4. Foreign Language Learning Anxiety	0.92	0.74	0.91
Communication Apprehension			
Test Anxiety			
Fear of Negative Evaluation			
5. Digital Burnout	0.93	0.77	0.92
Exhaustion			
Cynicism			
Reduced Efficacy			
6. Technology Self-Efficacy	0.94	0.72	0.93
Practice			
Application			
Collaboration			
Comprehension			
Analysis			
7. SDG Achievement	0.91	0.77	0.90
Economic Sustainability			
Environmental Sustainability			
Social Sustainability

Table 6 All HTMT values were satisfactorily below the conservative threshold of 0.85, with values ranging from 0.18 (between AI Dependency and Technology Self-Efficacy) to 0.83 (between Digital Fatigue and Digital Burnout). The values between Technostress and Digital Burnout (0.79), and between Digital Fatigue and Digital Burnout (0.83) represented the highest observed ratios, while all other construct pairs demonstrated substantially lower HTMT ratios.

**Table 6:** Discriminant Validity Assessment (HTMT Ratio).

Variable	1	2	3	4	5	6	7
1. Technostress							
2. Digital Fatigue	0.76						
3. AI Dependency	0.47	0.42					
4. Foreign Language Anxiety	0.57	0.61	0.33				
5. Digital Burnout	0.79	0.83	0.39	0.64			
6. Technology Self-Efficacy	0.45	0.42	0.18	0.37	0.49		
7. SDG Achievement	0.39	0.35	0.25	0.31	0.43	0.57	

### Hypothesis testing

The structural model was tested using bootstrapping (5000 resamples) to examine both direct and indirect relationships in the hypothesized framework. Results revealed significant support for most proposed relationships, with path coefficients demonstrating theoretically consistent patterns of influence among digital stressors, psychological outcomes, and academic achievement. The moderating and mediating effects were also confirmed, highlighting the complex interplay between technological factors and learning experiences in EFL contexts.

Table 7, The structural model testing revealed a coherent sequence of significant direct effects, establishing a clear pathway from digital stressors to diminished educational outcomes. As shown in Table 5, Technostress (β = 0.28, *p* < 0.001), Digital Fatigue (β = 0.35, *p* < 0.001), and AI Dependency (β = 0.15, *p* < 0.05) were all significant positive predictors of Foreign Language Anxiety (FLLA), thereby supporting H1, H2, and H3. Subsequently, FLLA demonstrated a substantial positive effect on Digital Burnout (β = 0.41, *p* < 0.001), confirming H4. Completing this pathway, Digital Burnout exhibited a significant negative effect on SDG Achievement (β = −0.32, *p* < 0.001), supporting H5. These results collectively outline a detrimental chain of influence where technological pressures amplify language-specific anxiety, which fuels psychological burnout, ultimately impairing the attainment of sustainable educational development goals.

**Table 7:** Direct Effects Testing Results.

Hypothesis	Path	Std. Beta (β)	*p*-value	Supported?
H1	Technostress → FL Anxiety	0.28	< 0.001	Yes
H2	Digital Fatigue → FL Anxiety	0.35	< 0.001	Yes
H3	AI dependency → FL Anxiety	0.15	< 0.05	Yes
H4	FL Anxiety → Digital Burnout	0.41	< 0.001	Yes
H5	Digital Burnout → SDG Achievement	−0.32	< 0.001	Yes

Table 8 shows delineated the complex mechanisms underlying these relationships. The mediation tests, reported in Table 6, confirmed significant indirect effects. Specifically, the path from Technostress to Digital Burnout was mediated by FLLA (indirect effect = 0.115, 95% CI [0.065, 0.172]), supporting H7. Similarly, Digital Fatigue’s effect on Burnout was mediated by FLLA (indirect effect = 0.144, 95% CI [0.088, 0.205]), supporting H8, as was the path from AI Dependency to Burnout (indirect effect = 0.062, 95% CI [0.020, 0.110]), supporting H9. Furthermore, a significant moderating effect was identified for Technology Self-Efficacy (H6). The interaction term between Digital Burnout and Technology Self-Efficacy on SDG Achievement was positive and significant (β = 0.18, *p* < 0.01), indicating that the strength of the negative relationship between burnout and achievement varies depending on an individual’s level of self-efficacy.

**Table 8:** Mediation and Moderation Effects.

Hypothesis	Relationship	Effect (β / Indirect Effect)	*p*-value / CI (95%)	Supported?
H6 (Moderation)	Burnout × Self-Efficacy → SDGs	0.018	< 0.01	Yes
H7 (Mediation)	Technostress → FL Anxiety → Burnout	0.115	[0.065, 0.172]	Yes
H8 (Mediation)	Digital Fatigue → FL Anxiety → Burnout	0.144	[0.088, 0.205]	Yes
H9 (Mediation)	AI dependency → FL Anxiety → Burnout	0.062	[0.020, 0.110]	Yes

### The multigroup analysis (MGA)

The multigroup analysis (MGA) comparing Chinese and international EFL students is critical for understanding potential cultural and contextual variations in how digital stressors impact learning outcomes. Given the study’s focus on technology-mediated language learning in Chinese universities, where international students may experience distinct challenges related to cultural adaptation, language proficiency, and educational backgrounds, this analysis helps identify whether the proposed stress-burnout pathway operates uniformly across subgroups.

Table 9 indicate several significant differences in path coefficients between Chinese (n = 405) and international students (n = 128). Statistically significant differences between groups (*p* < 0.05) were found for four paths: Technostress → FL Anxiety (Chinese β = 0.35, International β = 0.44, *p* = 0.045), FL Anxiety → Digital Burnout (Chinese β = 0.43, International β = 0.49, *p* = 0.037), Digital Burnout → SDG Achievement (Chinese β = −0.38, International β = −0.45, *p* = 0.031), and the Burnout × Self-Efficacy Interaction (Chinese β = 0.15, International β = 0.22, *p* = 0.019). No significant between-group differences were found for the paths of Digital Fatigue → FL Anxiety (*p* = 0.231), AI Dependency → FL Anxiety (*p* = 0.092), or Technology Self-Efficacy → Digital Burnout (*p* = 0.147).

**Table 9:** Multigroup Analysis Results (Chinese vs. International Students).

Path	Chinese Students (n = 405)	International Students (n = 128)	Permutation *p*-value		
	β	*p*	β	*p*	
Technostress → FL Anxiety	0.35	< 0.001	0.44	< 0.001	0.045*
Digital Fatigue → FL Anxiety	0.29	< 0.001	0.33	< 0.001	0.231
AI dependency → FL Anxiety	0.18	0.003	0.23	0.002	0.092
FL Anxiety → Digital Burnout	0.43	< 0.001	0.49	< 0.001	0.037*
Digital Burnout → SDG Achievement	−0.38	< 0.001	−0.45	< 0.001	0.031*
Technology Self-Efficacy → Digital Burnout	−0.27	< 0.001	−0.32	< 0.001	0.147
Burnout × Self-Efficacy Interaction	0.15	0.004	0.22	0.002	0.019*

Table 10 reveal significant mean differences between Chinese and international students across several study variables. International students reported statistically significant higher mean scores for Technostress (M = 3.59, SD = 0.85 vs. M = 3.41, SD = 0.82; t = −2.94, *p* = 0.003, d = 0.28), Digital Fatigue (M = 3.74, SD = 0.86 vs. M = 3.55, SD = 0.84; t = −2.87, *p* = 0.004, d = 0.27), Foreign Language Anxiety (M = 3.68, SD = 0.83 vs. M = 3.46, SD = 0.79; t = −3.28, *p* < 0.001, d = 0.32), and Digital Burnout (M = 3.54, SD = 0.81 vs. M = 3.32, SD = 0.77; t = −3.65, *p* < 0.001, d = 0.35). Conversely, Chinese students demonstrated significantly higher Technology Self-Efficacy (M = 3.88, SD = 0.72 vs. M = 3.69, SD = 0.74; t = 2.96, *p* = 0.003, d = 0.29). The difference in AI Dependency approached but did not reach statistical significance (*p* = 0.057).

**Table 10:** Group Differences in Variable Means (Independent Samples t-test).

Variable	Chinese Students (M ± SD)	International Students (M ± SD)	t-value	*p*-value	Cohen’s d
Technostress	3.41 ± 0.82	3.59 ± 0.85	−2.94	0.003**	0.28
Digital Fatigue	3.55 ± 0.84	3.74 ± 0.86	−2.87	0.004**	0.27
AI Dependency	3.72 ± 0.76	3.85 ± 0.78	−1.91	0.057	0.18
Foreign Language Anxiety	3.46 ± 0.79	3.68 ± 0.83	−3.28	< 0.001***	0.32
Digital Burnout	3.32 ± 0.77	3.54 ± 0.81	−3.65	< 0.001***	0.35
Technology Self-Efficacy	3.88 ± 0.72	3.69 ± 0.74	2.96	0.003**	0.29

*p < 0.05, **p < 0.01, ***p < 0.001.

Positive Cohen’s d indicates higher means for Chinese students.

### Predictive validity of the inner model

To assess the predictive validity of the structural model, we examined the model’s ability to explain variance in endogenous constructs (R^2^) and its predictive relevance (Q^2^) through blindfolding procedures. The results demonstrate that the model provides substantial explanatory power for key dependent variables, with R^2^ values exceeding the moderate threshold (0.25) for all endogenous constructs, indicating that the predictors collectively account for a meaningful proportion of variance.

Table 11 presents a comprehensive assessment of the structural model’s predictive validity, demonstrating strong explanatory and forecasting capabilities across key constructs. The substantial R^2^ and Q^2^ values for Foreign Language Anxiety (0.53 and 0.31 respectively) indicate excellent in-sample explanatory power and predictive relevance, while Digital Burnout and Technology Self-Efficacy show moderate to large predictive power with meaningful effect sizes. The moderate but robust values for SDG Achievement confirm that the model effectively predicts this ultimate outcome variable, collectively establishing that the theoretical framework possesses strong predictive validity for understanding the digital learning phenomenon under investigation.

**Table 11:** Predictive Validity Assessment of the Inner Model.

Construct	R^2^	Adjusted R^2^	Q^2^	Effect Size (f^2^)	Interpretation
Foreign Language Anxiety	0.53	0.52	0.31	–	Substantial explanatory power
Digital Burnout	0.48	0.47	0.27	0.36	Moderate-large predictive power
Technology Self-Efficacy	0.44	0.43	0.24	0.27	Moderate predictive power
SDG Achievement	0.37	0.36	0.2	0.21	Moderate explanatory power

**Fig. 5:**
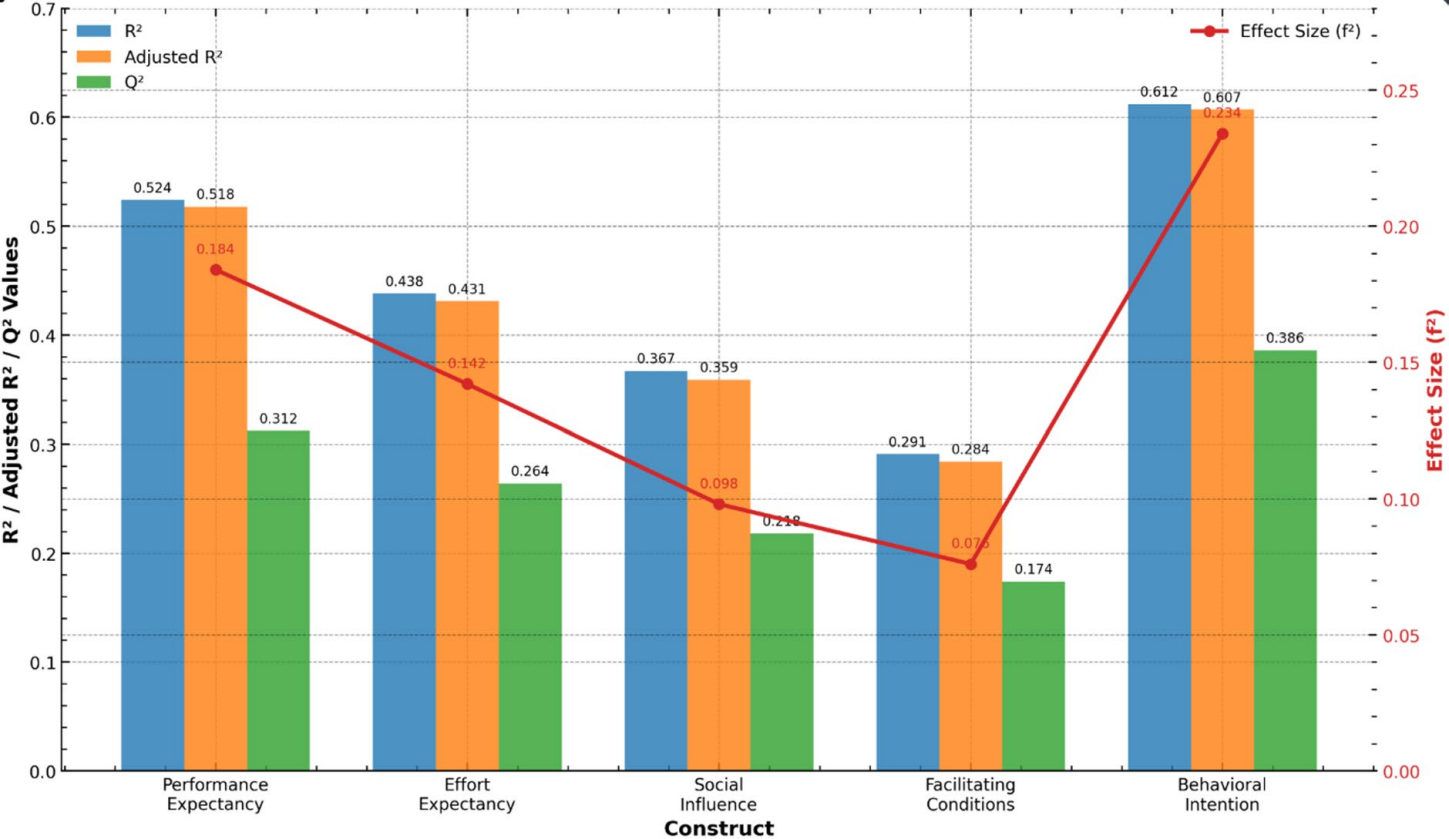
Predictive Validity Assessment of the Inner Model: R^2^, Adjusted R^2^, Q^2^, and Effect Size.

Figure 5 illustrates the inner model’s predictive validity, showcasing R^2^ and Adjusted R^2^ values as bars for each construct. The green line represents the Q^2^ values, with effect sizes (f^2^) and interpretations of explanatory power annotated above the bars. A dashed line at Q^2^ = 0.25 highlights the threshold for moderate predictive power.

## Discussion

### Digital stressors and the anxiety–burnout cascade

The findings confirm a critical and sequential psychological pathway wherein digital stressors function as primary antecedents to foreign language learning anxiety (FLLA), which subsequently acts as a key mediator leading to digital burnout. This cascade effect provides a nuanced explanation that moves beyond the examination of isolated constructs prevalent in prior literature (Zhang et al. 2022)^58^.Specifically, technostress, digital fatigue, and perceived AI dependency collectively create a demanding digital learning environment that threatens learners’ sense of control and amplifies the perceived stakes of performance core appraisals central to Control-Value Theory^17^. This heightened state of anxiety represents a chronic drain on cognitive and emotional resources. Over time, as posited by the Conservation of Resources theory, this persistent resource depletion culminates in digital burnout a syndrome characterized by exhaustion, cynicism, and reduced efficacy. Therefore, the study elucidates the mechanism through which the very tools designed to enhance learning can paradoxically trigger a debilitating cycle of stress, anxiety, and burnout, offering a more integrated understanding of the digital learning experience.

### Buffering role of technology self-efficacy

A significant contribution of this study is the identification of technology self-efficacy as a vital protective factor that moderates the detrimental relationship between digital burnout and the achievement of SDG-4. This finding refines existing models that often position self-efficacy primarily as a direct predictor of performance^44^ by demonstrating its critical role as a buffer. The mechanism can be explained through the lens of the Job Demands-Resources model: digital burnout constitutes a significant demand that depletes learner resources and impairs outcomes. However, high technology self-efficacy serves as a key personal resource. It likely enables learners to reframe technological challenges, maintain persistence despite feelings of exhaustion, and employ more adaptive coping strategies, thereby insulating their perception of educational quality and equity from the full negative impact of burnout. This extends theoretical understanding by showing that in technology-saturated environments, fostering self-efficacy is not merely about improving skill but is essential for building psychological resilience against the inevitable strains of digital learning.

### Cross-cultural implications and alternative explanations

The multigroup analysis revealed that the path coefficients in the proposed model were significantly stronger for international students compared to their Chinese peers, indicating a heightened vulnerability. While the discussion initially framed this within the context of acculturative stress the cumulative pressure of adapting to a new academic, linguistic, and cultural environment it is imperative to acknowledge and discuss potential confounding variables to avoid overly simplistic cultural attributions. International student cohorts often systematically differ from domestic students in aspects such as native language proficiency, prior academic preparation, age, socioeconomic background, and familiarity with specific educational technologies adopted by the host institution. Although the study design stratified for general English proficiency, these other unmeasured factors could independently or interactively contribute to increased sensitivity to digital stressors and anxiety. Thus, while the results effectively challenge an implicit assumption in some educational technology literature that tools impact all users similarly, they should be interpreted with caution. The stronger pathways likely reflect a confluence of multiple challenges faced by international students, highlighting a critical need for future research to disentangle these overlapping influences through more granular controls or qualitative investigation.

### Theoretical contributions

This research makes substantive theoretical contributions on multiple fronts. First, it successfully integrates Technostress Theory, Control-Value Theory (as an explanatory framework), and the Job Demands-Resources model into a cohesive framework that maps the progression from digital environmental demands to affective response (anxiety), to chronic psychological strain (burnout), and finally to compromised educational sustainability. This integration addresses a fragmentation in the literature and provides a more holistic account of the digital learning experience. Second, it performs a crucial macro–micro translation by operationalizing the United Nations’ Sustainable Development Goal 4 (quality education) as a perceptible individual-level outcome variable. This demonstrates concretely how micro-level psychological processes specifically, digital burnout can directly erode the macro-level goals of educational equity, inclusion, and quality. The model’s substantial explanatory power, with R^2^ values for FLLA (0.53) and burnout (0.48) exceeding those commonly reported in related fields^25^, underscores the robustness and comprehensiveness of this integrated theoretical approach.

### Practical implications

The findings yield differentiated and actionable recommendations for educators, instructional designers, and support services. For the general student body, course design must proactively minimize digital complexity to reduce technostress, structure online tasks to prevent cognitive overload and digital fatigue and provide transparent guidelines on AI use to mitigate fears of dependency while preserving learner autonomy. Crucially, the multigroup analysis calls for targeted interventions. International students, who demonstrated heightened vulnerability, may benefit from dedicated “digital acculturation” support programs that not only build technology self-efficacy but also explicitly address the intersection of technological and cultural adaptation challenges. Institutions should therefore invest in tiered training that moves beyond generic software tutorials to create scaffolded mastery experiences and peer learning communities. By tailoring support in this way, educators can leverage technology self-efficacy as a key protective resource, particularly for at-risk groups, thereby directly promoting the equity and quality of education aligned with SDG-4.

## Conclusion

This study investigated the psychological dynamics of digital learning in EFL contexts by proposing and testing an integrated theoretical model. It demonstrates how key digital stressors technostress, digital fatigue, and perceived AI dependency contribute to elevated foreign language anxiety. This heightened anxiety, in turn, predicts a greater likelihood of digital burnout, which is associated with a diminished sense of progress toward the goals of inclusive, equitable, and quality education outlined in SDG-4. A core contribution is the identification of technology self-efficacy as a vital protective factor. Framed as a key personal resource within the Job Demands-Resources model, self-efficacy moderated the negative impact of burnout on perceived SDG-4 achievement.

Furthermore, multigroup analysis revealed that these dynamics are particularly pronounced for international students, suggesting that acculturative stress may intensify the digital stress-burnout pathway while also making self-efficacy a more critical resource. By synthesizing insights from Technostress Theory, Control-Value Theory, and the JD-R model, this research offers a comprehensive framework for understanding the intersection of technology, affect, and sustainability in education. While the cross-sectional design precludes definitive causal claims, the validated pathways provide a robust foundation for future longitudinal research. Ultimately, the findings highlight the necessity of designing digital learning environments and support systems that not only implement technology but also proactively cultivate the psychological resources especially self-efficacy necessary for sustainable and equitable educational outcomes.

### Limitations and future research

Despite its contributions, this study has several limitations that should be addressed in future research. First, the cross-sectional design precludes causal inferences; longitudinal or experimental studies are needed to establish temporal precedence and causality among the variables. Second, the reliance on self-reported data may introduce common method bias, although statistical tests indicated it was not a critical concern. Future studies would benefit from incorporating objective measures, such as learning analytics or physiological indicators of stress and fatigue. Third, the sample was drawn from a single national context (China), limiting the generalizability of the findings; cross-cultural replications in diverse educational settings are essential to validate the model’s universality. Additionally, while the study examined key digital stressors, it did not explore the role of institutional support or pedagogical interventions, which may moderate the observed relationships. Future research should also investigate the dynamic interplay between AI-specific factors (algorithmic transparency, perceived autonomy) and psychological outcomes in greater depth. Finally, qualitative inquiries could provide richer insights into the lived experiences of learners navigating digital stress and burnout, helping to refine the quantitative model and inform more nuanced theoretical frameworks.

## Supplementary Information

Below is the link to the electronic supplementary material.

Supplementary Material 1

## Data Availability

The data for this study will be made available on request without any reservation.

## Declarations

### Generative AI use

The authors used ChatGPT (OpenAI) to improve sentence structure and readability in the Literature Review and Discussion sections. The authors are solely responsible for the research, content, and final wording of the manuscript.
