# Supplementary material for: Technostress, digital fatigue, and AI dependency as antecedents of burnout and SDG-4 achievement in EFL classrooms
Source: Sci Rep. 2026 Apr 1;16:15412. doi: 10.1038/s41598-026-45402-7 (PMC13184225; doi:10.1038/s41598-026-45402-7)
Supplement: Supplementary file 1 — Supplementary Material 1 [file 41598_2026_45402_MOESM1_ESM.docx]

**Questionnaire**

Strongly disagree (SD) = 1, Disagree (D) = 2, Neutral (N) =3, Agree (A) = 4, Strongly agree (SA) = 5

| **ITEM No** | **Statements** | **Strongly Disagree** | **Disagree** | **Neutral** | **Agree** | **Strongly agree** |  |
| --- | --- | --- | --- | --- | --- | --- | --- |
|  | **Adapted Version**  **Technostress** | | | | | | **Original Version**  **Technostress** |
|  | **Abilities-Demands Misfit (ADT)** | | | | | | **Abilities-Demands Misfit (ADT)** |
| ADT1 | I feel stressed by the need to constantly adapt to new digital tools or platforms in my EFL classes. | | | | | | I feel stressed to adapt to technology-enhanced learning. |
| ADT2 | I find it difficult to use digital learning tools for my English studies effectively because it requires more time and effort than I can often invest. | | | | | | I find it difficult to effectively use technology-enhanced learning due to my limited investment of time and effort. |
| ADT3 | I feel stressed because the technical demands of my digital EFL coursework exceed my current capabilities. | | | | | | I feel stressed to cope with the high demands of technology-enhanced learning with my current capability. |
| ADT4 | I find it hard to keep up with the frequent updates and new features of the language learning software or apps we are required to use. | | | | | | I find it hard to catch up with the constant updates of technology-enhanced learning with my current skillset. |
| ADT5 | I am pressured to change my current learning habits and preferences to meet the requirements of technology and AI-enhanced EFL instruction. | | | | | | I am pressured to change my current learning habit and preference to meet the requirements of technology-enhanced learning. |
| ADT6 | The use of technology in my EFL course increases my academic workload and pressures me to work harder. | | | | | | I am pressured to work harder due to technology-enhanced learning. |
| ADT7 | I am pressured to work faster in completing EFL tasks due to the pace set by technology-enhanced learning platforms. | | | | | | I am pressured to work faster due to technology-enhanced learning |
|  | **Needs-Supplies Misfit (NST)** | | | | | | **Needs-Supplies Misfit (NST)** |
| NST1 | I feel stressed as the digital/AI tools in my EFL classes are not useful in meeting my needs for better language proficiency and academic performance. | | | | | | I feel stressed as technology-enhanced learning is not useful in meeting my needs for better academic performance. |
| NST2 | I feel stressed as EFL learning technologies are not very relevant for the improvement of my language skills and SDG-4 related competencies. | | | | | | I feel stressed as technology-enhanced learning is not very relevant for the improvement of my study. |
| NST3 | I am irritated by the vast variety of digital tools, apps, and AI resources we are expected to use in EFL learning. | | | | | | I am not comfortable with the pervasive invasion of technology-enhanced learning in all aspects of my study. |
| NST4 | I am irritated by the vast variety of digital tools, apps, and AI resources we are expected to use in EFL learning. | | | | | | I am irritated by the vast variety of technology-enhanced learning. |
| NST5 | I feel stressed as the various forms of technology and AI in EFL complicate my study process and cause digital fatigue. | | | | | | I feel stressed as the various forms of technology-enhanced learning complicate my study. |
| NST6 | I feel stressed as the heavy reliance on technology and AI in my EFL courses disrupts my normal, effective study patterns. | | | | | | I feel stressed as the heavy reliance on technology-enhanced learning in my school disrupts my normal study pattern. |
|  | **Digital Fatigue** | | | | | | **Digital Fatigue** |
| DF1 | I feel exhausted after online classes | | | | | | I feel exhausted after online classes |
| DF2 | Excessive screen time is causing significant eye strain and visual stress. | | | | | | Excessive screen time is causing stress on my eyes. |
| DF3 | I often experience headaches or migraines linked to prolonged use of learning technologies and AI platforms. | | | | | | I often feel headache |
| DF4 | I get tired very quickly during online class | | | | | | I get tired very quickly during online class |
| DF5 | My sleep patterns have become decreased and disturbed due to the demands or blue light exposure. | | | | | | I am suffering from decreased and disturbed sleep |
| DF6 | After online classes, I tend to avoid social situations. | | | | | | After online classes, I tend to avoid social situations. |
|  | **Perceived AI Dependency** | | | | | | **Perceived AI Dependency** |
| PAID1 | I feel unprotected when I do not have access to AI daily. | | | | | | I feel unprotected when I do not have access to AI. |
| PAID2 | I’m concerned about the idea of being left behind in my tasks or projects if I do not use AI. | | | | | | I’m concerned about the idea of being left behind in my tasks or projects if I do not use AI. |
| PAID3 | I do everything possible to stay updated with AI to impress or remain relevant in my field | | | | | | I do everything possible to stay updated with AI to impress or remain relevant in my field. |
| PAID4 | I constantly need validation or feedback from AI systems to feel confident in my decisions | | | | | | I constantly need validation or feedback from AI systems to feel confident in my decisions. |
| PAID5 | I fear that AI might replace my current skills or abilities | | | | | | I fear that AI might replace my current skills or abilities. |
|  | **Foreign Language Learning Anxiety (FLLA)** | | | | | | **Foreign Language Learning Anxiety (FLLA)** |
|  | Communicative Apprehension | | | | | | Communicative Apprehension |
| CA1 | I feel quite sure of myself when I am speaking in my English class | | | | | | I never feel quite sure of myself when I am speaking  in my foreign language class. |
| CA2 | It frightens me when I do not understand what the teacher is saying. | | | | | | It frightens me when I don't understand what the  teacher is saying in the foreign language. |
| CA3 | I start to panic when I have to speak without preparation in English class. | | | | | | I start to panic when I have to speak without preparation in language class. |
| CA4 | I would be nervous while speaking English with native speakers. | | | | | | I would not be nervous speaking the foreign language  with native speakers. |
| CA5 | I get upset when I do not understand what the teacher is correcting. | | | | | | I get upset when I don't understand what the teacher  is correcting. |
| CA6 | I feel confident when I speak in English in class. | | | | | | I feel confident when I speak in foreign language class |
| CA7 | I feel very self-conscious about speaking English in front of other students. | | | | | | I always feel that the other students speak the foreign  language better than I do. |
| CA8 | I get nervous and confused when I am speaking in my English class. | | | | | | I get nervous and confused when I am speaking in my  language class |
| CA9 | I get upset when I do not understand what the teacher is saying in English. | | | | | | I get nervous when I don't understand every word the  language teacher say. |
| CA10 | I feel overwhelmed by the number of rules you have  to learn to speak a foreign language. | | | | | | I feel overwhelmed by the number of rules you have  to learn to speak a foreign language. |
| CA11 | I would probably feel comfortable around native speakers of English. | | | | | | I would probably feel comfortable around native  speakers of the foreign language |
|  |  | | | | | |  |
|  | **Test Anxiety** | | | | | | **Test Anxiety** |
| TA1 | I tremble when I know that I am going to be called on in English class. | | | | | | I tremble when I know that I'm going to be called on  in language class |
| TA2 | It would bother me at all to take more English classes. | | | | | | It wouldn't bother me at all to take more foreign lan-  guage classes. |
| TA3 | During English classes, I find myself thinking about the things that have nothing to do with the course. | | | | | | During language class, I find myself thinking about  things that have nothing to do with the course. |
| TA4 | I am usually at ease during tests in my classes. | | | | | | I am usually at ease during tests in my language class |
| TA5 | I worry about the consequences of failing my English class. | | | | | | I worry about the consequences of failing my foreign  language class |
| TA6 | I better understand why some people get so upset over English class. | | | | | | I don't understand why some people get so upset over  foreign language classes. |
| TA7 | In English class, I can get so nervous that I forget things I know. | | | | | | In language class, I can get so nervous I forget things  I know |
| TA8 | Even if I am well prepared for English class, I feel anxious about it. | | | | | | Even if I am well prepared for language class, I feel  anxious about it |
| TA9 | I often feel like I am going to my English class. | | | | | | I often feel like not going to my language class |
| TA10 | I can feel my heart pondering when I am going to be called on in English class. | | | | | | I can feel my heart pounding when I'm going to be  called on in language class. |
| TA11 | The more I study for an English test, the more confused I get. | | | | | | The more I study for a language test, the more con-  fused I get. |
| TA12 | I feel pressure to prepare very well for the English class. | | | | | | I don't feel pressure to prepare very well for language  class. |
| TA13 | English class moves so quickly that I worry about getting left behind. | | | | | | Language class moves so quickly I worry about getting left behind. |
| TA14 | I feel more tense and nervous in my English class than in my other classes. | | | | | | I feel more tense and nervous in my language class than in my other classes. |
| TA15 | When I am on my way to English class, I feel very sure and relaxed. | | | | | | When I'm on my way to language class, I feel very sure and relaxed. |
|  | **Fear of Negative Evaluation** | | | | | | **Fear of Negative Evaluation** |
| FNE1 | I worry about making mistakes in English class. | | | | | | I don't worry about making mistakes in language class. |
| FVE2 | I keep thinking that the other students are better at English than me. | | | | | | I keep thinking that the other students are better at  languages than I am. |
| FNE3 | It embarrasses me to volunteer answers in my English class. | | | | | | It embarrasses me to volunteer answers in my language class |
| FNE4 | I am sorry that my English teacher is ready to correct every mistake I make. | | | | | | I am afraid that my language teacher is ready to correct every mistake I make |
| FNE5 | I always feel that the other students speak English better than I do. | | | | | | I always feel that the other students speak the foreign  language better than I do |
| FNE6 | I am sorry that the other students will laugh at me when I speak English. | | | | | | I am afraid that the other students will laugh at me  when I speak the foreign language |
| FNE7 | I get nervous when the English teacher asks questions that I have not prepared in advance. | | | | | | I get nervous when the language teacher asks questions which I haven't prepared in advance. |
|  | **Digital Burnout in Learning** | | | | | | **Digital Burnout in Learning** |
|  | **Digital Aging** | | | | | | **Digital Aging** |
| DA1 | Due to constant digital engagement for EFL learning, I have an attention deficit. | | | | | | I have attention deficit |
| DA2 | I think that I will lose my mind one day from the pressures of digital EFL learning. | | | | | | I think that I will lose my mind one day |
| DA3 | When using digital tools for language learning, I sometimes feel like my mind gets blurred. | | | | | | I sometimes feel like my mind gets blurred |
| DA4 | I feel stressful because of the constant demands of the digital EFL learning environment. | | | | | | I feel stressful |
| DA5 | Either my hand or my body aches as a result of constantly typing and checking messages for my EFL coursework. | | | | | | Either my hand or my body aches as a result of constantly writing and checking messages. |
| DA6 | I started to think that I have symptoms of depression linked to my digital EFL studies. | | | | | | I started to think that I have symptoms of depression |
| DA7 | A feeling of loneliness dominates me in my technology-mediated EFL learning journey. | | | | | | A feeling of loneliness dominates me. |
| DA8 | I am confused about my academic status due to the overload of digital EFL learning. | | | | | | I am confused about my statue. |
| DA9 | I feel restricted by the digital systems and platforms required for my EFL classes. | | | | | | I feel restricted. |
| DA10 | I cannot establish a balance between the real world and the virtual world of EFL learning. | | | | | | I cannot establish balance between the real world and the virtual world |
| DA11 | I spend long periods of time in the virtual world with digital devices for EFL tasks. | | | | | | I spend long periods of time in the virtual world with digital devices. |
| DA12 | Because of digital fatigue from EFL studies, I speak and look around less. | | | | | | I speak and look around less |
|  | **Digital Deprivation** | | | | | | **Digital Deprivation** |
| DD1 | I feel uneasy when I do not have an internet connection or I am offline and cannot access my learning materials. | | | | | | I feel uneasy when I do not have internet connection or I am offline |
| DD2 | I always think about which notification I just received and what is happening on the learning platforms. | | | | | | I always think about which message I just received and what is happening. |
| DD3 | I feel disconnected from my studies when I do not have my digital devices (phone, tablet, computer, etc.) with me. | | | | | | I feel naked when I do not have my digital devices (phone, tablet, computer etc…) with me |
| DD4 | I check my learning platforms, emails, and class messages all the time. If I don't, I feel weird or anxious. | | | | | | I check my tweets, facebook account, e-mails, messages all the time. If I don't, I feel weird or anxious |
| DD5 | I feel powerless when I do not have an internet connection and cannot proceed with my digital work. | | | | | | I feel powerless when I do not have an internet connection, or I am offline. |
| DD6 | I feel most afraid of losing or forgetting my phone because it contains all my learning access. This thought disturbs me. | | | | | | I fell most afraid of losing or forgetting my phone. This thought disturbs me. |
|  | **Emotional Exhaustion** | | | | | | **Emotional Exhaustion** |
| EE1 | I feel exhausted due to the virtual and digital worlds of my education. | | | | | | I feel exhausted due to virtual and digital worlds. |
| EE2 | I almost feel nothing about academic events and situations around me because of digital overload. | | | | | | I almost feel nothing about events and situations around me. |
| EE3 | I have become intolerant of and desensitized to the people around me due to stress from digital learning. | | | | | | I have become intolerant of and desensitized to the people around me |
| EE4 | I have become impatient as a result of the frustrations with learning technologies. | | | | | | I have become impatient. |
| EE5 | I have become quick-tempered because of constant digital demands in my language studies. | | | | | | I have become quick-tempered. |
| EE6 | I think that my relationships and communications with people have been weakened by my preoccupation with digital tasks. | | | | | | I think that my relationships and communications with people have been weakened. |
|  | **Technology Self-Efficacy** | | | | | | **Technology Self-Efficacy** |
|  | Practice | | | | | | Practice |
| P1 | I can learn to use a new digital language learning platform or AI tool step by step | | | | | | I can assemble a robot step by step. |
| P2 | I know how to use the core features and tools of digital/AI applications for EFL learning. | | | | | | I know how to use the tools of making a robot. |
| P3 | I can troubleshoot basic technical problems (e.g., connectivity, software glitches) when using EFL learning technologies. | | | | | | I can build up a circuit for a robot. |
| P4 | I can successfully complete a task or assignment using the required digital or AI tool in my EFL class. | | | | | | I can make a robot. |
|  | **Application** | | | | | | **Application** |
| APP1 | I can propose ideas for using digital tools or AI to solve specific EFL learning problems (e.g., improving vocabulary, practicing pronunciation). | | | | | | I can propose the ideas of using robots to solve problems. |
| APP2 | I can apply my knowledge of EFL learning technologies to enhance my daily study routines. | | | | | | I can apply robotics knowledge in my daily life. |
| APP3 | I can make effective use of a specific digital platform or AI assistant to solve a language-related problem. | | | | | | I can make use of a robot to solve a problem. |
|  | **Collaboration** | | | | | | **Collaboration** |
| COLLA1 | I can discuss easily with peers how to use digital tools or AI applications for collaborative EFL projects. | | | | | | I can discuss easily with peers how to make robots. |
| COLLA2 | I can present my ideas clearly in my EFL class when explaining how I used a technology to complete work. | | | | | | I can present my ideas clearly in a robotics classroom. |
| COLLA3 | I can express my opinions freely in my EFL class regarding the use of different learning technologies. | | | | | | I can express my opinions freely in a robotics classroom. |
|  | **Comprehension** | | | | | | **Comprehension** |
| COMP1 | I am clear about the core concepts and purposes behind the digital/AI tools we use in EFL learning. | | | | | | I am clear about the learning concepts of robotics. |
| COMP2 | I can answer teachers’ questions about the functionality or educational value of the technologies used in my EFL class. | | | | | | I can answer teachers’ questions in a robotics classroom. |
| COMP3 | I can link the use of specific EFL learning technologies with concepts from other subjects or real-world applications. | | | | | | I can link robotics concepts with other learning subjects. |
|  | **Analysis** | | | | | | **Analysis** |
| ANA1 | I can evaluate several digital or AI-based solutions for solving a given EFL learning task. | | | | | | I can evaluate several robotic solutions for solving a problem. |
| ANA2 | I can demonstrate an idea or language concept by effectively using an appropriate digital tool or AI feature. | | | | | | I can demonstrate an idea by making a robot. |
| ANA3 | I can think about a challenge in digital EFL learning from different angles to find the best technological approach. | | | | | | I can think of a robotics problem from different angles. |
|  | **Sustainable Development Goals (SDG)** | | | | | | **Sustainable Development Goals (SDG)** |
|  | **Social Sustainability** | | | | | | **Social Sustainability** |
| SOC1 | In education, equal opportunities should be offered to all individuals (women/men, rich/poor, etc.). | | | | | | Equal opportunities should be offered to individuals in society (women/men, rich/poor, race/religion etc.). |
| SOC2 | As a learner, I believe environments should be created for all individuals to enable lifelong learning. | | | | | | For all individuals in society, environments should be created to enable the individual to learn lifelong. |
| SOC3 | Societies should provide individuals with integrating social services (such as nurseries, assistance foundations) to support educational participation. | | | | | | Individuals should be provided with integrating and enhancing social services (such as nurseries,  shelter homes, social assistance foundations etc.). |
| SOC4 | Access to quality education and health services should be provided to all individuals in society. | | | | | | Access to education and health services should be provided to all individuals in society. |
| SOC5 | Individuals should be provided with environments, including digital learning spaces, where they feel safe. | | | | | | Individuals should be provided with environments where they feel safe while living |
| SOC6 | In our globalized classrooms, the interaction of cultures should be supported and developed. | | | | | | Interaction of cultures in society should be supported and developed. |
| SOC7 | Society must take responsibility to keep the well-being of individuals and families, including students, above a minimum. | | | | | | The society must take responsibility to keep the well-being of individuals and families above the  minimum. |
| SOC8 | Urbanization should protect the soul and body health of the society, ensuring access to healthy learning environments. | | | | | | Urbanization (city, town, etc.) should be to protect the soul and body health of the society. |
| SOC9 | The work of organizations involved in activities for a sustainable and equitable educational environment should be supported. | | | | | | The work of governmental and non-governmental organizations involved in activities for the  sustainable environment should be supported. |
|  | **Economic Sustainability** | | | | | | **Economic Sustainability** |
| ECO1 | Individuals should shop according to their desires, but as future professionals, we must also consider sustainable consumption. | | | | | | Individuals should shop in the direction of their desires and wishes without regard to their needs. |
| ECO2 | We must use current economic resources with conservation, thinking about future generations. | | | | | | We must use current economic resources with conservation, thinking about future generations |
| ECO3 | Debt for development should consider long-term economic balances. | | | | | | Debt to be made for development should be made considering economic balances |
| ECO4 | Economic policies should be able to reduce poverty and differences in income distribution. | | | | | | Economic policies should be able to reduce poverty and differences in income distribution. |
| ECO5 | Economic development should be planned to prevent unemployment. | | | | | | Economic development should be planned to prevent unemployment. |
| ECO6 | Economic policies should be shaped by sustainable production. | | | | | | Economic policies should be shaped by sustainable production |
| ECO7 | Economic policies should be shaped so as not to destroy natural resources. | | | | | | Economic policies should be shaped so as not to destroy natural resources. |
| ECO8 | I believe that livestock, agricultural and industrial production should NOT be focused only on short-term profit (e.g., use of GMOs, hormonal animals). | | | | | | Livestock, agricultural and industrial production should be focused on applications that will generate  high profits in the short term (use of GMO products, hormonal animals etc.) |
| ECO9 | For economic investments, environments where life and property safety are provided must be established. | | | | | | For economic investments, environments where life and property safety are provided must be  established |
| ECO10 | For economic development, sustainable sectors should be emphasized. | | | | | | For economic development, non-production sectors should be emphasized. |
| ECO11 | The production of sustainable high-tech products for economic development should be supported. | | | | | | The production of high-tech products for economic development should be supported. |
| ECO12 | Sustainable investments in agriculture and livestock sectors should be supported. | | | | | | Investments in agriculture and livestock sectors should be supported for economic development. |
| ECO13 | Research and development (R&D) studies for sustainable economic development should be supported. | | | | | | Research and development (R&D) studies for economic development should be supported. |
|  | **Environmental Sustainability** | | | | | | **Environmental Sustainability** |
| ENV1 | Any intervention that damages natural life must be punished for the continuation of biological diversity. | | | | | | Any intervention that damages natural life (wrong use of pesticide, prohibited hunting, etc.) must  be punished for the continuation of biological diversity |
| ENV2 | The use of public transportation at short distances helps to maintain atmospheric equilibrium. | | | | | | The use of public transportation at short distances does not help to maintain atmospheric equilibrium |
| ENV3 | I think that vehicles with the least impact on degradation of ecological balance should be preferred. | | | | | | I think that vehicles with the least impact on degradation of ecological balance should be preferred  when buying one. |
| ENV4 | Energy-saving products should be preferred to use energy sources longer. | | | | | | Energy saving products should be preferred in order to use energy sources for a longer time. |
| ENV5 | The use of renewable energy sources needs to be widespread to leave a livable world. | | | | | | The use of renewable energy sources needs to be widespread to leave a livable world. |
| ENV6 | Every individual, including students like me, has a responsibility to protect existing resources for future generations. | | | | | | Every individual has responsibility to protect existing resources (water, air, soil etc.) for future  generations to survive ecological problems |
| ENV7 | Industrial establishments should take cautions to protect environmental health and prevent pollution. | | | | | | Industrial establishments should take cautions to protect environmental health and prevent pollution  of natural resources. |
| ENV8 | Green areas cannot be dispensed with for urbanization and industrialization. | | | | | | Green areas can be dispensed with for urbanization and industrialization. |
| ENV9 | To leave a greener world, responsibility for afforestation is the responsibility of each individual. | | | | | | In order to leave a greener world for future generations, responsibility for afforestation and the  protection of the trees is the responsibility of each individual. |
| ENV10 | I think that each individual has responsibilities in the process of recycling wastes. | | | | | | I think that each individual has responsibilities in the process of recycling wastes so that the raw  material resources can be used by future generations. |
| ENV11 | Wastes should be separated and reused so that raw material sources can be used by future generations. | | | | | | Wastes should be separated according to their characteristics and reused, so that raw material  sources can be used by future generations. |
| ENV12 | I think that a lot can be done individually to prevent global climate change. | | | | | | I think that nothing can be done individually to prevent global climate change. |
| ENV13 | I think global warming poses a serious threat to the future if cautions are not taken. | | | | | | I think global warming poses a serious threat to the future of our world if cautions are not taken. |
| ENV14 | I think that our ecological footprint should be minimized for the continuation of the world's livability. | | | | | | I think that ecological footprint should be minimized for the continuation of the world's livability. |

Thank you for your time and honest response.
